# Protein-Based Drug-Delivery Materials

**DOI:** 10.3390/ma10050517

**Published:** 2017-05-09

**Authors:** Dave Jao, Ye Xue, Jethro Medina, Xiao Hu

**Affiliations:** 1 Department of Physics and Astronomy, Rowan University, Glassboro, NJ 08028, USA; jaod07@students.rowan.edu (D.J.); xuey5@rowan.edu (Y.X.); medina99@students.rowan.edu (J.M.); 2 Department of Biomedical Engineering, Rowan University, Glassboro, NJ 08028, USA; 3 Department of Biomedical and Translational Sciences, Rowan University, Glassboro, NJ 08028, USA

**Keywords:** protein biopolymer, drug delivery, controlled release, silk, collagen, elastin, keratin

## Abstract

There is a pressing need for long-term, controlled drug release for sustained treatment of chronic or persistent medical conditions and diseases. Guided drug delivery is difficult because therapeutic compounds need to survive numerous transport barriers and binding targets throughout the body. Nanoscale protein-based polymers are increasingly used for drug and vaccine delivery to cross these biological barriers and through blood circulation to their molecular site of action. Protein-based polymers compared to synthetic polymers have the advantages of good biocompatibility, biodegradability, environmental sustainability, cost effectiveness and availability. This review addresses the sources of protein-based polymers, compares the similarity and differences, and highlights characteristic properties and functionality of these protein materials for sustained and controlled drug release. Targeted drug delivery using highly functional multicomponent protein composites to guide active drugs to the site of interest will also be discussed. A systematical elucidation of drug-delivery efficiency in the case of molecular weight, particle size, shape, morphology, and porosity of materials will then be demonstrated to achieve increased drug absorption. Finally, several important biomedical applications of protein-based materials with drug-delivery function—including bone healing, antibiotic release, wound healing, and corneal regeneration, as well as diabetes, neuroinflammation and cancer treatments—are summarized at the end of this review.

## 1. Introduction

New discoveries in medicine and the way different ailments should be treated create a need for more sophisticated methods of drug delivery. Traditionally, the therapeutic efficacy of biomolecular drugs, such as proteins and oligonucleotides, is often limited by their short half-lives due to proteolysis and renal clearance [1,2]. Drugs in blood circulation could be rapidly filtered in the kidney and cleared via the reticuloendothelial system (RES) before reaching the target site. For drugs administered through injection, the vasculature can provide a direct path to the site of disease or take detours where the drug can be eliminated. However, these drugs have to be administered at a high dosage and frequency to perfuse and circulate through the vasculature, finally diffusing into the tissue interstitium, to achieve the desired therapeutic effect [3]. While the oral route for administering drugs is the most convenient, safe and widely accepted method, these bioactive compounds must be insoluble in the stomach to avoid substantial losses due to acid and pepsin in the stomach and pancreatic enzymes in the small intestine [4]. Once it reaches the intestine, drugs must be dissolvable and absorbed through the intestinal mucosa [3]. These above traditional methods are not suitable for chronic and persistent medical conditions requiring drug-delivery systems that are non-toxic, long-term, and controlled to deliver the correct dosage at the correct time. Therefore, there is an overwhelming need for mechanically stable polymer materials as drug-delivery carriers to encapsulate therapeutics from degradation and clearance, while sparing the rest of the body from excess toxicity. Polymer materials can be easily processed into different shapes and structures including films, microcapsules, microspheres, nanoparticles, micelles, gels, and fibers. With the application of polymer carrier systems, therapeutics and biomolecular proteins can protect itself against the harsh environment of the gastrointestinal tract and provide efficient site-specific delivery avoiding the risk of significant side effects and immunological responses.

Interests in protein-based biopolymers for drug delivery have increased in recent years. Compared to synthetic polymers, they have advantages of being water-soluble, biocompatible, biodegradable and non-toxic [4]. Many natural animal proteins such as keratin, collagen, elastin, and silk are relatively inexpensive and sustainable. They are easily derived from their natural sources and simple to process under mild conditions. These proteins have been studied extensively, having high biocompatibility and favorable structural properties for various biomedical applications. Drug-delivery methods using these protein polymers form an essential part of the pharmaceutical applications with release behavior, degradation profile, and mucoadhesive nature. Plant-based proteins, such as zein derived from maize (corn), have also proven to be a very attractive drug-delivery carrier that has enhanced interaction with the biological environment, absorption, and retention time. Zein proteins have been modified and tested in formats of different polarities based on pH via electrostatic interactions or hydrophobic bonds with anti-microbial agents and anticancer drugs for controlled release [5]. Other plant proteins (such as soy protein and wheat gliadin) are also frequently explored for various drug-delivery applications, which can be used to deliver proteins, peptides, DNA, and vaccines [2]. With animal and plant proteins, their respective hydrolysates and small peptides can be recycled from agricultural, aquatic (fisheries) and animal (poultry and meat) sectors and be directly employed in high-priority fields such as biomedical engineering and pharmaceutics [6].

In this review, we will first discuss the structure and potential applications of these natural protein-based polymers. Next, we will discuss the fabrication of various materials from these proteins and their drug-release efficacy. Using these natural protein polymers as an excipient for transdermal, nasal, ocular and oral drug delivery, we will also discuss in detail the effects of drug particle size and density and their binding capacity.

## 2. Protein Materials

**Figure 1:**
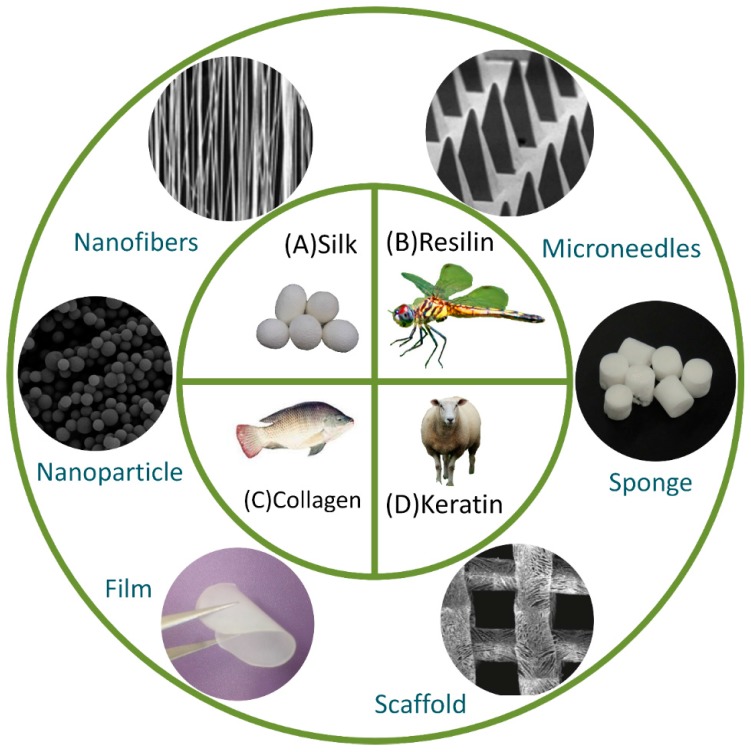
Various proteins and their possible sources under research for drug-delivery applications: (**A**) silk from *Bombyx mori* cocoons; (**B**) resilin from dragonfly; (**C**) collagen from fish scales; (**D**) keratin from goat hairs. These proteins can be fabricated into various drug-delivery vehicles such as films, sponges, gels, fibers, nanoparticles and microneedles. (Reproduced with permission from Reference [7,8,10,11], ACS Publications and Elsevier.)

Many fibrous protein materials such as keratin, collagen, elastin and silk have been widely used in drug-delivery research (Figure 1). Since protein materials share similar properties, they can be processed in similar manners [7,8,9].

### 2.1. Keratin

Keratin is a fibrous protein found in the integumentary system of humans and animals. It can be derived from the outer layer of human skin and the epidermal appendages of animals such as feathers, hair, hooves, horns, nails, scales, and wool [6]. Compared to petroleum-based polymers, keratin is very viable and cost-effective option for biomedical applications. About 95% of pure keratins can be derived from recycled wool with the rest being other components such as hydrocarbons [12]. At the molecular level, keratin can have three different configurations: *α*-, *β*- and *γ*-keratin. The *α*-keratin is an intermediate filament protein that has an *α*-helix structure consisting of four right-handed *α*-helices intertwined to form a protofibril. It has a molecular weight ranging from 40 to 70 kDa and a low sulfur amino acid content of 1.5–2% w/w which can form disulfide bridges from adjacent chains. The *β*-keratin is also an intermediate filament protein but has a *β*-sheet structure with amino acids rich in glycine, alanine, serine, lysine, histidine and tryptophan with a molecular weight ranging from 11 to 22 kDa. The structure is stabilized by hydrogen since no cysteine thiol groups are present [6]. The *γ*-keratin is a matrix protein that has an amorphous structure containing a large amount of cysteine, glycine and tyrosine. It has a molecular weight ranging from 11 to 28 kDa and a high sulfur amino acid content of 4–8% w/w which can form the high degree of intermolecular and intramolecular disulfide bonds. Based on the crosslinking cysteine residues, hard and soft keratins can be generated for mechanical, thermal, and chemical stability [12]. Unlike collagen and elastin, wool is a cheap source of keratin, which is the by-product of the textile industry. Keratin biomaterials extracted from wool and human hairs are biocompatible, biodegradable, nontoxic and very tunable. Further, keratin can contain cell adhesion motifs, RGD (Arg-Gly-Asp) and LDV (Leu-Asp-Val), which mimic the sites of cellular attachment for the development of drug-delivery vehicles [13]. It can be prepared into various forms such as gels, films, fiber, and sponge for various drug-delivery applications, wound dressings and neural tissue-repairing applications.

### 2.2. Collagen

Collagen is a protein imperative to the structural integrity of tissues and cell growth in vertebrates and other organisms [14]. It is an animal-based protein with three polypeptide chains found in various connective tissues. Accounting for 30% of all proteins in mammals, collagen has various types in most of the extracellular matrix. Type I collagen, in particular, is a major component of different extracellular matrices present in skin, arteries, bone, and corneas [14]. Collagen can be used as a highly biocompatible and versatile protein material. It can be processed into various forms suitable for drug delivery (such as hydrogels, microparticles and films) [15]. One challenge in treating collagen proteins is the heat. To make collagen water soluble, a relatively high amount of heat is necessary to process. Such heat is not suitable for withholding drugs and other substances in collagen films, microparticles, and other materials. As a result, using other solvents such as organic acids can be employed. Areas of applicability for collagen-based materials include bone healing and cancer treatment [15].

Collagen is also a dominant candidate material for corneal regeneration due to its high biocompatibility. Since the cornea is mainly made up of type I collagen, it is a promising drug-delivery biomaterial for corneal repair. Even though collagen fulfills a variety of physiological functions and is very durable in vivo, extracted collagen is easily degradable in vitro, due to the dissociation of natural crosslinks during isolation and purification process [16]. To reduce the rate of enzymatic and hydrothermal degradation, collagen can be chemically crosslinked to provide improved mechanical stability. There are several ways to chemically crosslink collagen molecules, but they are divided into two categories: bi-functional and amide-type. Bifunctional reagents, such as glutaraldehyde, genipin, polyethylene glycol diacrylate (PEG-DBA), and hexamethylene diisocyanate, are used to bridge amine groups of lysine or hydroxylysine residues of collagen polypeptide chains and other natural protein-based polymers [16,17]. However, a major handicap of these chemical crosslinking reagents is the potential cytotoxicity or chromogenic effect of residual molecules or compounds. While genipin and carbodiimide (amide-type) do not have cytotoxicity, most crosslinking agents have the potential to be released into the natural environment during degradation [18]. By selecting and employing safe crosslinking modification methods to collagen, drug stability and retention times can be increased.

### 2.3. Elastin

Similar to collagen, elastin is a protein within the extracellular matrices of different flexible tissues [19]. It is one of the most stable proteins in the body that can be stretched and relaxed more than a billion times and remain extremely insoluble. Elastin is a heavily crosslinked structure with beta-spiral secondary structure, making up a major component in elastic fibers [19]. While natural elastin is insoluble, there is also research into soluble elastin derivatives, such as human tropoelastin [19,20]. Elastin-like polypeptides (ELP) can also be synthesized. They are biopolymers composed of repetitious chains of the valine, proline, glycine, and unknown (Xaa) amino acids (e.g., VPGXG) from natural elastin protein sequence [21]. With low critical solution temperature and phase transition behavior, elastin composites are feasible materials for stimulus-responsive controlled drug release and vascular stents [22]. Functionalized elastin nanoparticles, microparticles, and macromolecular carriers can also be controlled genetically, resulting in control over size and sequence. The potential diseases/conditions of interest using elastin materials include osteoarthritis, cancer, and type II diabetes [22].

Elastin is also a thermoresponsive protein that provides structure and maintains elasticity of many types of tissues and organs in the human body such as skin, blood vessels, lung, and connective tissues. Various forms of thermoresponsive elastin, such as animal-derived soluble elastin, recombinant human tropoelastin (rhTE), and elastin-like polypeptides (ELPs), have been synthesized and utilized to engineer promising synthetic tissue scaffolds [23]. ELPs are polypeptides derived from the hydrophobic domains of elastin. They are widely used as thermoresponsive units in biomaterials due to the presence of a sharp soluble-to-insoluble phase change at a specific transition temperature. These elastin-derived molecules can be assembled linearly or become star-shaped using lysine amino acids as branching and terminal units with 1–3 pentameric repeats between each branch [24]. The ELP transition temperatures can also be tuned using different forms of the sequence, Xaa-Pro-Gly-Xaa-Gly (XPGXG), where *X* represents different amino acids, to adjust the lower critical solution temperature (LCST) behavior [23]. With the ability to change from random coil to *β*-turn conformation upon heating through the transition temperatures, ELPs can demonstrate temperature-dependent flow and retention with the opening and closing of pores when processed into various forms of three-dimensional porous hydrogels, elastomeric films, and electrospun scaffolds. Being able to adjust pore size and stress-stiffness, ELPs can enhance cell growth and mediate mesenchymal stem cell fate.

### 2.4. Silk

Silk is a protein of long historical use in biomedical applications, such as tissue and ligament repair, nerve regenerators, and artificial blood vessels [25]. Silk fibroin protein is mainly derived from silkworm cocoons and spider threads with insoluble beta-sheet crystal structures. Different silkworm silks have been used for drug-delivery applications including *Bombyx mori*, *Tussah*, and *Eri* silks [26]. Silk proteins can be prepared in various ways, such as films [27], 3D porous scaffolds [28], and micro and nanoparticles [29], with controlled degradation rates. Silk is an excellent material for drug-delivery applications due to its mild processing conditions and aids in reducing cost. With desirable biocompatibility and controllable biodegradability, silk-based materials make ideal long-term drug-eluting depots. Silk-controlled release properties are defined by two primary methodologies: diffusion of substance payload and solubilization/degradation of the silk material [28]. A thin silk film with programmable solubility rate, for example, can release the desired substance as the film degrades within the designated area [28].

Compared to naturally spun silkworm fibers, spider dragline silks have many attractive physical properties that include significantly higher tensile strength and elasticity. The development of silk fibers having the properties of spider silks is of keen interest. However, the generation of a spider silk-manufacturing process faces serious problems through spider farming, since spiders are quite territorial by nature. Therefore, standard recombinant silk protein production platforms were employed. Thus far, only small quantities of artificial spider silk can be produced due to the inability to successfully assemble spider silk proteins into fibers with native-sized recombinant silk protein (250–320 kDa) [30]. While native-sized spider silk protein can be favorably expressed in metabolically engineered *Escherichia coli*, the recombinant proteins can also yield lower molecular weight versions, which leads to inferior fiber properties [31]. To overcome these limitations, Teulè et al. using recombinant DNA introduced a unique genetic engineering vector called ‘PiggyBac’ [30]. These vectors are pieces of DNA known as a transposon taken from spiders that can insert itself into the genetic machinery of silkworms. The silk fibers produced by these transgenic silkworms were a combination of chimeric silkworm and spider silk proteins. The genetically engineered silk proteins produced are an extremely stable composite material with improved elasticity and strength as native dragline spider silk fibers. By using recombinant DNA, gene manipulations into current commercial silkworm production can be a potentially viable solution for large-scale production of engineered protein fibers with customizable properties of strength and elasticity that can exceed properties of native silkworm silk. This biotechnological approach can lead to a broad range of mechanical properties, optimized for specific biomedical and drug-delivery applications, such as fine suture materials, bandages, or scaffolds for tendon and ligament repair in addition to strong and lightweight structural fabrics.

### 2.5. Resilin

Resilin is an elastomeric protein first found existing in jumping insects’ cuticles of many species [32]. It contains coiled amino acid chains that form a flexible and elastic network structure when the di- and tri-tyrosine links are crosslinked [32]. Under high-frequency deformation, contraction and extension, the dityrosine crosslinked protein structures possess extraordinary resilience and long-range reversible elasticity. Resilin also exhibits high extensibility, low stiffness, efficient energy storage and extraordinary resilience, which enables some jumping insects to jump many times’ their body length. The resilin’s rubber-like elasticity also possess excellent biocompatibility and mechanical properties, which can be used for a broad range of biomedical applications such as drug delivery and tissue scaffolds [32,33,34].

### 2.6. Corn Zein

Many types of plant-based proteins have been used for drug-delivery applications. Zein is a major plant-based storage protein rich in prolamine found in the endosperm of the corn kernel [35]. It is a by-product from the processing of maize corn present in corn gluten meal and from the manufacturing of ethanol during the wet and dry milling processes [36]. Zein has a molecular weight of about 40 kDa and is rich in glutamine, proline, alanine, and leucine residues [5]. It is grouped into four classes, *α*-, *β*-, *γ*- and *δ*-zein, with varying molecular weights and modes of extraction [36]. The *α*-zein consists of 70–85% of the total zein with molecular weights ranging from 22 to 24 kDa with a predominant triple super-helix protein structure; followed by 10–20% of *γ*-zein consists with molecular weights ranging from 18 to 27 kDa. The remaining minor fractions consist of 1−5% of *β*-zein with a molecular weight of 17 kDa high in methionine and 1−5% of *δ*-zein with molecular weight of 10 kDa [37]. All zein fractions have hydrophobic and hydrophilic domains but zein is frequently considered to be a hydrophobic protein due to its insolubility in water and solubility in ethanol, acetone, and acetylacetone. The non-polar helical interiors of the zein protein have glutamine-rich turns and loops that allow it to self-assemble into particles and layers [35]. With successive helical segment folding in an antiparallel arrangement, zein can exhibit chemical and thermoplastic properties that are heat and pH stable [37]. Other excellent material properties of zein include biodegradability, mechanical resistance and water barrier ability, making it attractive in applications such as drug delivery and coatings in food and pharmaceuticals [35]. Also, zein has shown to form aggregates and entrap solutes like drugs or amino acids which make it an excellent matrix material for sustained release.

## 3. Fabrication Methods of Devices Based on Biopolymers

Once the raw protein materials are prepared, there are different ways to process them, and the method ultimately used is determined by their delivery applications (some key examples have been summarized in Table 1). Drug stability, release kinetics, and method of administration are all factors that affect the final form of the material.

### 3.1. Films and Coatings

Films are thin layers of a particular substance. They are flexible and can cover vast areas. These two properties open avenues for drug delivery on various surfaces using films. Wound dressing, for example, requires a material that can match the flexibility of the skin and the pressure forced onto it. Using protein-based films infused with drug treatment can be continuous while reducing the risk of infection from multiple treatments and drug admissions.

Silk films can be prepared by several steps. First silk cocoons or raw fibers are degummed to separate the sericin from the silk fibroin fibers [67]. The degummed fiber is then dried, followed by dissolution in lithium bromide or other organic solvents. The resulting silk solution is dialyzed for further purification to obtain a silk aqueous solution. This new solution can be then cast into various molds or areas to form silk films [67]. Microparticles and nanoparticles containing a desired drug or substance can be incorporated into silk films in the solution stage for controlled drug-release applications.

Collagen films are fabricated in a similar manner to silk films. The process begins with obtaining dried collagen [68]. The dried collagen is dissolved in an acidic solution and then neutralized. Crosslinkers, such as acrylamide, are added to facilitate crosslinking of collagen protein chains [69]. The resulting solution can be cast into films for drug-delivery applications. The addition of other substances can increase the range of use of the films, such as providing collagen films antimicrobial properties [69].

Elastin protein and ELPs can be fabricated into films as well. HFIP (hexafluoro-2-propanol), ddH_2_O, or acetic acid are appropriate solvents for making elastin and ELP solutions [68]. The elastin solutions are cast and set at room temperature for the period aforementioned. As the solvent evaporated, films will be produced within 48 h [68]. Incorporating ELP-drug composites into the films will allow for additional controlled drug release from the fabricated films [68].

### 3.2. Particles and Spheres

Particles for drug-delivery applications are typically on the micro- and nano-scale. Microparticles are particles with diameters between 0.1 and 100 μm, while nanoparticles measure between 1 and 100 nm in diameter. Encapsulating drugs and other substances into particles of such size will increase release capabilities in a targeted tissue or organ. Using protein-based particles will add the biocompatibility necessary to utilize microparticles and nanoparticles in controlled drug release [70].

Silk nanoparticles can be generated from different solvents such as acetone desolvation [28]. Acetone desolvation involves rapidly introducing aqueous silk fibroin solution to acetone. After drying, water-insoluble nanoparticles can be separated from the solution [71]. Applying an electric field to nanoparticles can also physically produce silk microspheres of 2–3 μm in diameter. Silk nanoparticles, when exposed to a weak electric field, were found to be able to aggregate into microspheres around the positive electrodes [71].

**Figure 2:**
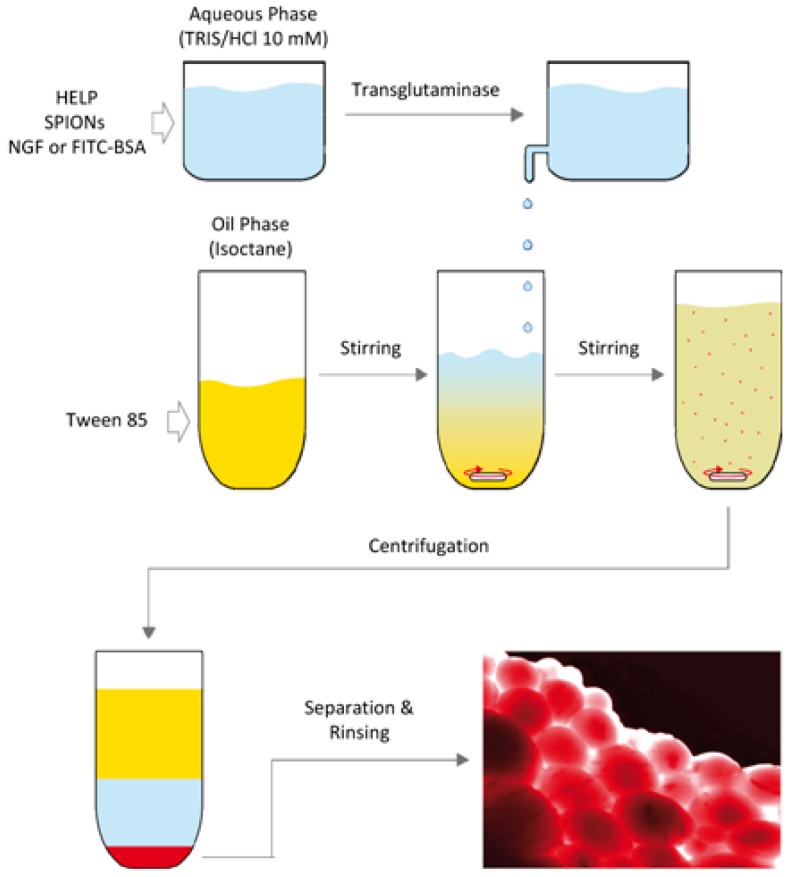
Preparation for the magnetic human elastin-like polypeptide (HELP) microparticles with various controlled drug-delivery applications. (Reproduced with permission from Reference [72], John Wiley and Sons).

Elastin-based magnetic ELP microparticles can be prepared through a water-in-oil emulsion. Introducing magnetic nanoparticles to the system, Cifani et.al found their magnetic properties can be transferred into the ELP microparticles [72]. Human elastin-like polypeptide (HELP) microparticles can also be prepared using the emulsion mentioned prior and a subsequent enzymatic crosslinking procedure [72]. A typical process for producing magnetic HELPs can be seen in Figure 2. Drug molecules are introduced into the aqueous phase in Figure 2, yielding magnetic, drug-loaded ELP microparticles.

**Figure 3:**
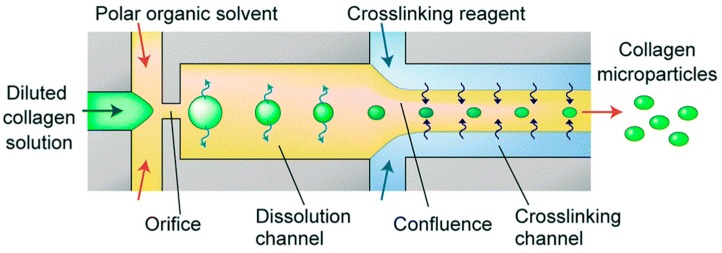
Microfluidic schematic for producing collagen microparticles. (Reproduced with permission from Reference [73], The Royal Society of Chemistry).

Collagen microparticles can be fabricated by two methods: membrane emulsification and the use of microfluidic devices [73]. The membrane emulsion method uses a membrane of uniform pore size and low pressure to produce particles of uniform size [74]. A collagen solution mixed with acetic acid is pumped through a membrane, into a continuous methyl acetate phase [73]. The solution pushed through the membrane was added dropwise to the methyl acetate. Centrifugation was then employed to separate the microparticles from the methyl acetate solution. Microfluidic devices were also used to produce microparticles. Figure 3 depicts the process for the fabrication of collagen microparticles using microfluidics. A diluted collagen solution is first fed through the opening of the device [73] and then introduced into a polar organic solvent (acetic acid). The mixture is finally mixed continuously with the crosslinking reagent (a glutaraldehyde solution) to form the particles [73]. The resulting microparticles are collected at the exit of the device, with a diameter of about 20–50 μm.

### 3.3. Hydrogels

Hydrogels are networks used in facilitating the controlled release of cells and other bioactive molecules [75]. Their three-dimensional design aids in the controlled release of molecules such as proteins, antibodies, and drugs. Hydrogels are sometimes infused with nanoparticles to further their uses in drug-release applications.

To form collagen hydrogels, a collagen solution is first prepared by dissolving dried collagens in an acidic solution [69]. The solution is then mixed with monomers (HEMA) and crosslinking agent (MBA) and neutralized before producing the hydrogels. Ammonium persulfate can be then be added to initiate crosslinking of the collagen within 12 h [69]. Additional collagen techniques such as plastic compression and vitrification can also be applied to collagen gels to form films. Placing the gels solution in Petri dishes and allowing the reaction to occur gives collagen fibrils the necessary time to reorganize and crosslink during solvent evaporation.

Unlike collagen, silk hydrogels can be prepared using physical methods without additional crosslinkers. This is due to the formation of beta-sheet crystals, which can act as crosslinkers in the hydrogel. To begin silk aqueous solution is prepared [75], then the silk solution is mixed and vortexed gently in an ethanol solution or directly sonicated in ultrasound, resulting in a physically crosslinked hydrogel system. By using an ethanol solution with drug-containing silk particles, the resulting hydrogel can contain the particular drug or substance of interest [75].

### 3.4. Microneedles

Microneedles can be employed as a method of transdermal drug delivery [10,76]. They can be utilized as a painless alternative to traditional hypodermic needles. Microneedles penetrate skin across the stratum corneum, giving patients a feeling of slight pressure with no pain [77]. Furthermore, sites of microneedle injection showed faster rates to reaching maximum blood flow when compared to topical applications.

**Figure 4:**
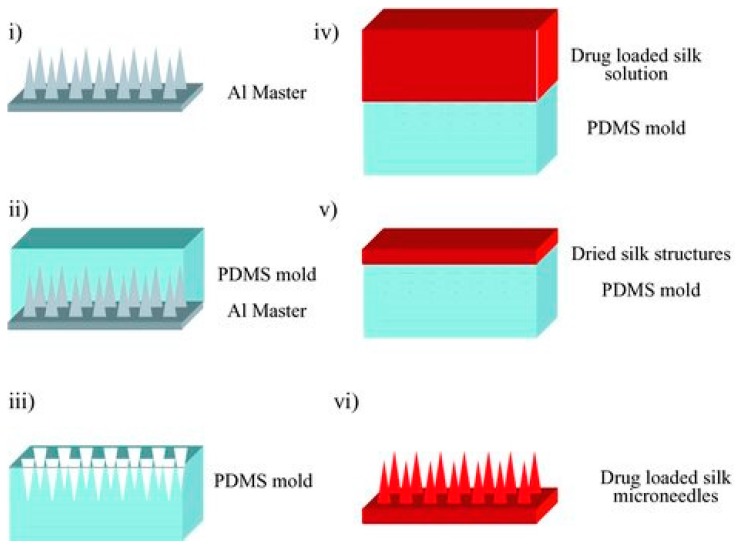
Schematic of silk microneedle fabrication for drug delivery. (Reproduced with permission from Reference [78], John Wiley and Sons).

Silk-based microneedles have been designed for controlled substance release with relatively mild processing conditions [28]. Controlling drug-release rates is dependent on the secondary structure and beta-sheet crystallinity of the silk materials [28], which can be adjusted by various post-processing procedures. One method of producing silk microneedles is provided in Figure 4. PDMS is first cast over an aluminum mold to create PDMS molds in the proper microneedle shape. The silk-drug solutions are then cast over the PDMS mold and dried. The silk layer is finally separated from the PDMS, resulting in silk-drug microneedles [78].

### 3.5. Composite Materials

#### 3.5.1. Keratin Composites

Medicated wound dressing-composite scaffolds have been developed using freeze-drying techniques. These solid sponge scaffolds based on keratin (K), fibrin (F) and gelatin (G) obtained show highly interconnected pores with macroporous morphology through SEM analysis. Mimicking extracellular matrix with cell-binding motifs in the keratin and fibrin can enhance NIH 3T3 fibroblast and keratinocyte growth, and adhesion can be seen in vitro fluorescence images. The efficiency of KFG-composite scaffolds have been studied and tested using mupirocin as the model drug. The drug-loaded scaffold showed a gradual, sustained drug release, which suggested that the scaffold could be used as an active antibacterial wound-dressing biomaterial in skin tissue regeneration [79].

Also in tissue regeneration, De Guzman et al. have studied the intermolecular surface and bulk interaction properties of reduced keratin proteins (kerateines) with gold and bone morphogenetic protein 2 (BMP-2) as potential drug carriers [79]. To investigate the gold to kerateines interactions, the group chemisorbed thiol-rich kerateines onto gold substrates to form a 2 nm rigid layer for surface plasmon resonance analysis. The gold substrate showed irreversibly link to the free thiols of kerateines forming strong intermolecular covalent disulfide bonds, which can lead to longer-lasting gels. To study the electrostatic forces between negatively charged keratins and positively charged growth factors, the binding and release kinetics of BMP-2 from the kerateines’ network are examined by electrostatic or coulombic interactions in various pH and salt concentrations. These findings contribute to the understanding of the release kinetics of BMP-2-conjugated kerateines systems, which can potentially be employed in bone repair and regeneration as drug-delivery vehicles [79].

Through thiol–ene click chemistry, keratin graft poly(ethylene glycol) (keratin-g-PEG) copolymer nanoparticles can be synthesized [80]. Due to the amphiphilicity and the thiol groups of the graft copolymers, the nanoparticles can self-assemble with PEG chains and keratin as the core in aqueous solutions. The disulfide bonds can crosslink the core of the nanoparticles on the keratin backbones. With cleavable glutathione (GSH) crosslinks, the efficiency of the drug-delivery system has been evaluated using doxorubicin hydrochloride salt (DOX·HCl) as the model drug. The work concluded that the release of the loaded DOX is dependent on the concentration of GSH at the cellular level. The nanoparticles also showed excellent DOX-loading capacity of 18.1% (w/w) and accelerating release into the nucleus of the cells with the dual triggerable release properties of GSH and trypsin. With the controllable and responsive release of the loaded DOX, cells can efficiently internalize the DOX making it a promising carrier for intracellular drug delivery for cancer therapy and other drug-delivery applications [80].

Like other natural polymers, keratin promotes favorable cell interactions and degradable porous non-toxic scaffold for regenerative medicine. Han et al. developed alkylated kerateine hydrogels impregnated with rhBMP-2, rhIGF-1, and ciprofloxacin, for the differentiation of stem cells toward osteogenic lineage [81]. The scaffold was synthesized with an iodoacetamide alkylation (or “capping”) of cysteine residues on reductively extracted keratin (kerateines). Unlike the oxidatively derived keratin (keratose) that cannot form disulfide crosslinks, kerateines can provide a more stable crosslinked hydrogel network. Employing alkylation, the levels of disulfide crosslinking in keratin hydrogels can be modulated providing controllable rates of gel erosion and therapeutic agent release. The modification process of kerateines did not lead to increased cytotoxicity in MC3T3-E1 pre-osteoblasts and maintained the ability of cells to attach to the material at levels greater than collagen. The release of therapeutic agents appears to follow the rate of keratin hydrogel erosion with loss of disulfide crosslinking. The alkylated kerateine hydrogels provided good cell attachment and proliferation as while as controlled delivery, leading to drug concentrations appropriate for bone and tissue-engineering applications [81].

#### 3.5.2. Elastin Composites

For thermo-targeted chemotherapy of hyperthermic tumor margins, thermosensitive ELP-based diblock biopolymers have been developed containing functional poly-glutamic/aspartic acid blocks for drug conjugation [82]. The efficiency of ELP-based polymers was tested using geldanamycin (GA) as the model drug. Even though clinical trials of GA have not shown promise due to off-target toxicity and poor formulation design, this study stated that conjugation of geldanamycin (GA) with ELP-based polymer could be used for the thermo-targeted drug-delivery agents of the tumor margins and inhibition of heat shock protein 90 (HSP90), an essential molecular chaperone of several potent pro-oncogenic pathways. The ELP-based polymer-GA conjugates demonstrated high drug loadings and tunable thermo-responsiveness. Thermal precipitation of the biopolymer-GA conjugates at hyperthermic isotherms T > 40 °C provide active targeting at the tumor site while the pH-sensitive drug, GA, is released within the acidic tumor microenvironment. This avoids systemic toxicity and off-target effects, which presents a novel platform for anti-cancer treatment [82].

For gene delivery, tunable hollow ELP spheres (~100–1000 nm) were developed by Dash et al. Using the self-assembly property and slight positive charge of ELP, the group fabricated the hollow spheres using polystyrene (PS) beads as a template. Permeation studies of the polyplex (~70 μg pDNA/mg of hollow sphere) were carried out using plasmid DNA (pDNA) as the model drug [83]. To provide stability, the ELP spheres were crosslinked with microbial transglutaminase (mTGase) following the removal of the PS beads. The addition of pDNA into polyplex resulted in higher loading efficiency than that of self-assembled solid particles and controlled release triggered by protease and elastase. Moreover, polyplex-loaded hollow spheres also yielded higher luciferase expression by providing protection against endosomal degradation. Throughout this process, surface functional groups were well maintained. The technique is straightforward and efficient allowing ELP hollow spheres to be produced in large quantities. Designing advanced ELP hollow spheres with the ability to couple targeting ligands can provide exciting new opportunities in gene delivery applications [83].

For tumor-specific delivery and controlled drug release, thermosensitive ELP-grafted dipalmitoylphosphatidylcholine (DPPC)-based liposomes were developed by Kim et al. using a lipid film hydration method [84]. The group designed an ELP with a mild hyperthermia-mediated trigger using an *αvβ*3 integrin targeting moiety that induces target-specific endocytosis. Doxorubicin (DOX), an anticancer drug, was used as the model drug. Upon mild hyperthermia, 75% to 83% DOX was able to be released from the thermosensitive ELP at 42 °C and 45 °C. Using an ammonium sulfate-gradient method, the efficiency of cRGD-targeted and ELP-modified DOX-encapsulated liposomes (RELs) were studied. Since cyclic arginine-glycine-aspartic acid (cRGD) is overexpressed in the angiogenic vasculature and tumor cells, RELs showed an 8- to 10-fold overexpression in U87MG and HUVEC cells, which were higher than that of non-targeting liposomes. These highly specific RELs can be guided and activated using currently available external heat-generating devices such as ultrasound or radiofrequency [84].

#### 3.5.3. Collagen Composites

To be applied as drug-eluting implants or a drug-release system, McMaster et al. developed a highly biocompatible and porous collagen-based (CAC) scaffold coated with an alginate polymer [9]. The layer-by-layer collagen scaffold was assembled using a cryogenic plotting system. The efficiency of the drug-delivery system was evaluated using rhodamine B as the model drug. The system exhibited an initial burst release regulated by the porosity of the CAC scaffolds, followed by a constant drug release controlled by the volume percentage of alginate coated on the collagen scaffold. The CAC scaffolds have a Young’s modulus of 30 MPa, which is nine times higher than pure collagen scaffolds. With a porosity of 88%, biological function can be maintained. Osteoblast-like cells (MG63) seeded on CAC scaffolds have shown to proliferated and migrated into the interior of the scaffolds. The CAC scaffolds can be tuned for long-term therapeutic applications in tissue engineering [9].

For dentistry applications, glutaraldehyde crosslinked type-I collagen gels were developed by Barbaresso et al. [85] using a freeze-dried technique. Drug permeation studies were carried out on the 3D collagen matrices using niflumic acid as the model drug. Niflumic acid, an anti-inflammatory drug, was embedded into the collagen sponges using glutaraldehyde as the crosslinking agent. The addition of crosslinking agents resulted in the formation of collagen matrices with constant niflumic acid release rate. The crosslinked collagen gels showed reduced swelling and enzymatic degradation. Since the presence of niflumic acid did enhance water absorption, a non-Fickian kinetic mechanism was proposed for drug release. The released niflumic acid percent can be tuned by adjusting the amount of crosslinking agent, thus allowing the optimum release of the desired amount of drug in relation to the application site and therapeutic recommendations [85].

For topical drug delivery applications, antibiotic-loaded collagen (coll)-containing hydrogel films have been fabricated by mixing degraded collagen with synthetic monomers, such as acrylamide (AAm) and 2-hydroxy ethyl methacrylate (HEMA) [69]. Antibiotics, such as gallic acid (GA) and naproxen (NP), were then loaded into the crosslinked composites of p(coll-co-AAm) and p(coll-co-HEMA) hydrogel films to test its efficacy. The drug kinetics studies were carried out in vitro. Linear drug releases were observed in both GA-loaded p(coll-co-AAm) and NP-loaded p(coll-co-HEMA) composite films obtaining up to 32 h and 36 h release, respectively. As other studies have concluded, higher amounts of active agents could be increased by crosslinking collagen. This also provides additional advantages, such as better mechanical strength and absorption. Moreover, metal nanoparticles such as Ag and Cu can be added within these hydrogel films to further enhance the antimicrobial characteristic against known common bacteria such as *Escherichia coli*, *Bacillus subtilis*, and *Staphylococcus aureus*. The investigation showed that antibiotic-loaded collagen hydrogel films could be readily prepared and used as potential wound- and/or burn-dressing materials for drug-delivery and healing purposes [69].

Nanoparticles based on biopolymers or peptides can also be fabricated to have targeted and controlled release. Anandhakumar et al. developed collagen peptide (CP) for the preparation of chitosan (CN) nanoparticles via ionic gelation method by co-precipitation of CN and CP [86]. The CPCN nanoparticles are formed through hydrogen bonding and electrostatic interactions. They are pH-responsive and stable under physiological conditions. The efficiency of the drug-delivery system was evaluated using doxorubicin (DOX) hydrochloride as the model drug. The unique CPCN nanoparticles showed biphasic release with a burst release in the first 20 h, followed by sustained release over 7 days. In response to the extracellular pH of the tumor environment, enhanced delivery of anti-tumor drugs was also seen with 68% controlled release at pH 7.4 and 89% release at pH 1.5. With cell viability studies, DOX-loaded NPs showed excellent anti-proliferative characteristics against HeLa cells with favorable biocompatibility with NIH 3T3 fibroblast cells. Therefore, protein-based NPs have a high potential for encapsulation and release of anti-tumor drugs in the area of cancer drug delivery [86].

## 4. Factors to Control Drug-Delivery Efficiency

Delivery efficiency is one of the important factors in drug delivery. Protein materials with drug-delivery function need to guarantee that the drug can maintain a continuous release over a period of time at a certain therapeutic level. For poorly soluble drugs in particular, maintaining controllable drug-delivery efficiency is vital. Proper delivery efficiency can optimize the drug absorption and the cure effectiveness. It has been studied that the controllable drug delivery can be achieved through altering the molecular weight or monomer of the protein-polymer system or changing the size, porosity and morphology of the protein-material matrix. The drug-release efficiency may also vary due to the shape of the material, such as ellipsoid, disc or rods, as well as the delivery molecules. In this section, we will discuss the drug-delivery efficiency in the case of molecular weight, particle size, particle shape, morphology, and porosity of protein polymers.

### 4.1. Molecular Weight 

**Figure 5:**
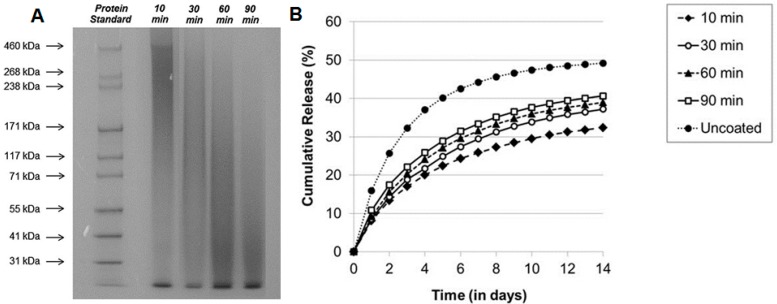
(**A**) SDS-PAGE analysis of silk with different degumming times (10 min, 30 min, 60 min and 90 min); (**B**) Cumulative release of rifampicin from silk gel films capped with a single layer of silk prepared using varied degumming times. (Reproduced with permission from Reference [27], John Wiley and Sons).

Natural silk fibroin materials, featuring good biocompatibility and biodegradability, have been sufficiently studied as a promising drug-delivery material. Pritchard et al. [87,88] reported that the drug-release rate could be controlled by changing the molecular weight of silk (Figure 5). To process raw silk fibers into desired drug-delivery materials, it is necessary to remove the hydrophilic sericin proteins, which functions as the glue coated on hydrophobic fibroin fibers, through degumming. When subjected to thermal and alkaline conditions during the degumming procedure, the molecular weight of silk fibroin fibers can be controlled by the degumming time. Thereby, the drug-release rate can be controlled by adjusting the duration of degumming, as shown in Figure 5 [27]. With the degumming time increasing, the molecular weight of silk fibroin materials will decrease, which may decrease the crystallinity and molecular chain length, and reduce physical crosslinks of silk materials. Besides the molecular weight, the chain length also has an impact on the drug diffusion through the silk matrix. A correlation between degumming time and silk molecular weight has been thoroughly studied by Pandit et al. [89] and Yamada et al. [90]. The drug-release behavior can be affected by both the diffusion of the drug through the silk-polymer matrix and the degradation of the matrix. Thereby, both factors can be manipulated through controlling the molecular weight of the silk-polymer matrix. Indigo carmine, rifampicin, reactive red 120 and azoalbumin particles have been used to study the relation between drug-release profile and the molecular weight of silk polymer [27]. It was found that the drug release increased while the molecular weight decreased (with longer degumming time). A similar study was also conducted by Fang et al. [91], which confirmed that reducing the molecular weight of silk in silk hydrogels can increase the drug-release rate qualitatively.

### 4.2. Nanoparticle Size

**Figure 6:**
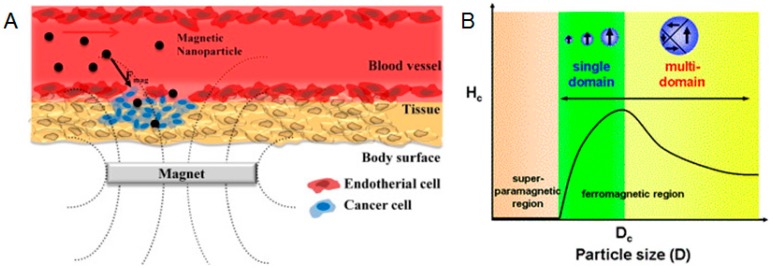
(**A**) Schematic of a magnetic nanoparticle-based drug-delivery system; (**B**) Plot of the magnetic coercivity (Hc) vs. the size of particle for drug delivery. (Reproduced with permission from Reference [98], Elsevier, and Reference [99], The Royal Society of Chemistry).

The drug load and release efficiency of protein nanoparticles can be largely influenced by the size distribution. Particle size is an important factor associated with biocompatibility [92]. By altering the nanoparticle size and controlling the size distribution, nanoparticles have the ability to penetrate the physiological barrier, which can affect the release efficiency and therapeutic impact [93]. In addition, synthesizing nanoparticles below their critical size can induce their magnetic (superparamagnetic) properties, which can be potentially used as an attractive drug-delivery vehicle for tumor cure [94,95,96]. In a report by Lu et al. [97], the group showed how mesoporous silica nanoparticles (MSNs) of about 50 nanometers could maximize cellular. This size factor can also be applied to magnetic particles delivered by biopolymers [98,99], as shown in Figure 6A, and the relationship between the magnetic coercivity and the particle size is shown in Figure 6B. While increasing the size of the particles, the distribution of nanoparticles circulated to the liver and spleen would increase [97]. Particle size can also affect the efficiency of nanoparticles extravasated from the vasculature. Therefore, maintaining good control of the nanoparticles size can overcome limits to certain tissue or organ where the traditional drug-delivery method cannot access, especially for the brain system.

The focus of osteoinductive biomaterials for bone tissue-engineering applications has recently been directed towards composite components that facilitate biomaterial integration, bone repair, and regeneration. Foo et al. have designed and developed a novel bioactive material that combines silk fibroin proteins with silica particles [100]. This unusual pairing can restore tissue structure and function at the implanted site by providing immediate structural support while remaining flexible. Unlike hydroxyapatite (HA) minerals which lack biological activity and low durability, silica is osteoinductive and has a morphological structure that can bear the load of hard tissue. Mieszawska et al. investigated the effect of silk-silica composites with human mesenchymal stem cells (hMSCs) subjected to osteogenic differentiation [101]. The study showed stimulated osteogenic cell growth and gene expression. With the addition of silk, smaller and more uniform silk-silica composites could be prepared with a diameter of 24 nm to 2 μm. This synergistic effect can decrease the size and amount of silica content used toward bone tissue regeneration.

### 4.3. Morphology and Shape

**Figure 7:**
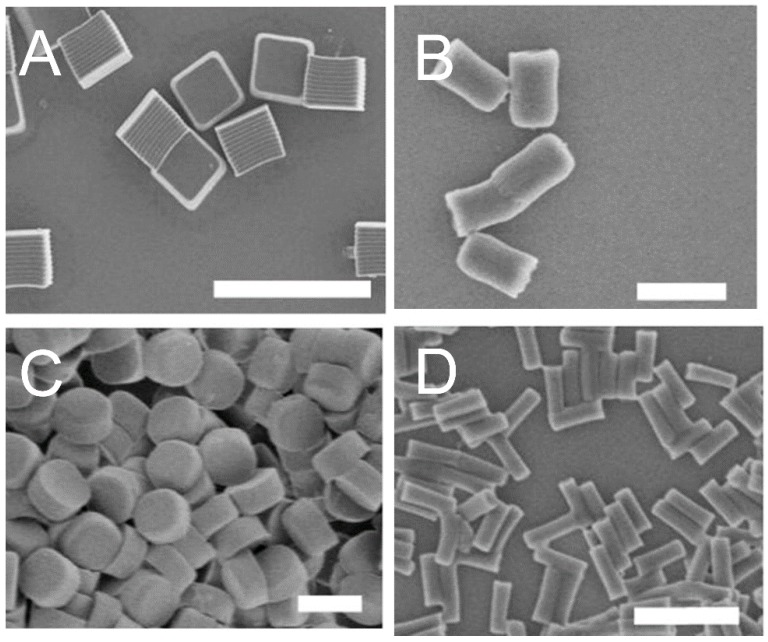
Micrographs of PRINT particles varying in both size and shape. The scale bars for (**A**) is 20 μm; (**B**–**D**) is 1 μm. (Reproduced with permission from Reference [103], Copyright (2008) National Academy of Sciences, U.S.A.).

Nanoparticle morphology and shape are important factors that influence the drug-delivery efficiency. Nanoparticles can take various form of morphology from a simple sphere, rod, or sheet to wormlike structures through different chemical and physical fabrication processes, as shown in Figure 7 [102,103,104]. The drug-release efficiency can be controlled by changing the geometry, coating thickness, porosity, or manipulating the crystallinity of the protein drug carriers [27]. Studies have been conducted to tailor the particle morphology through changing the monomer ratios of the copolymers and the pH of the solvent. Almería et al. [105] have controlled different morphologies of PLGA microparticles using an electrospray drying method. The morphology of the drug carrier particles were characterized by scanning electron microscopy (SEM) and transmission electron microscopy (TEM). To show how particle shape can affect the orientation, Champion et al. [106] illustrated that flexible disk-shaped red blood cells with a diameter less than 10 µm could pass through the spleen. However, if spherical-shaped nanoparticles were used, a diameter less than 200 nm could be used to pass through the spleen avoiding contact forces along vessel walls and other flow properties [106]. To achieve higher drug loading and eluting, drug-delivery vehicles can also be modified to possess large pore volumes and surface areas. For efficient drug delivery, a structure’s height, diameter and thickness and its ultimate shape should be studied to better tailor drug-release efficacy.

### 4.4. Porosity

Altering the porosity of a material is a common way to control drug delivery. Since surface area and permeability play an important part in drug-delivery vehicles, both the drug-loading capacity and release rate are directly associated with the porosity, pore size and pore interconnectivity of the material. Increasing the porosity percentage can enhance degradation and increase drug release in a polymer matrix, largely by the surface area to volume ratio. In order to achieve rapid drug delivery with a low percentage of pores, Itokazu et al. [107] developed an antibiotic-loaded hydroxyapatite ceramic scaffold with uniformly accessible pores, interlinked pore system, and enlarged macropores. Scaffold porosity may play an important role in determining drug-release kinetics [108,109].

## 5. Biomedical Applications

### 5.1. Bone Healing

Bone is an area of concentrated cellular and molecular organization, and any damage to the bone is considered a bone injury [110]. Bone tissue is capable of reconstruction following the time of injury. However, conditions exist such as osteoporosis resulting in weakened and brittle bones. Furthermore, bone is an area difficult to treat with drugs requiring heavy blood circulation [22]. The protein materials listed above with the various forms have been widely used to aid in bone healing. By providing access to drugs, stimuli, and other substances, protein-based drug delivery is part of the future of bone repair and bone strengthening.

For example, ELP depots can provide drug delivery not easily reached through blood circulation [22]. With a long half-life, the depots can be sustained for an extended period of time. These depots act as reservoirs of drugs and other substances favorable to treatment. The depots would be comprised of drug-loaded nanoparticles [22]. Taking advantage of the long half-life, the ELP depots can remain until sufficient drug has been released to induce adequate healing.

Collagen-based microparticles can also be employed for use in bone tissue regeneration [111]. To determine applicability, collagen-composite microparticle-based scaffolds were compared with non-collagen containing scaffolds [111] to demonstrate the statistical differences between the collagen and non-collagen models regarding degradation, cytocompatibility, porosity, and Young’s modulus. Furthermore, new studies are being conducted to incorporate growth factors, antimicrobial substances, and other materials with collagen-based microparticles to heighten bone regeneration capabilities [111].

Silk, too, can be employed for bone tissue repair [112]. Silk scaffolds, similar to collagen, have the mechanical stability to be loaded into high-stress areas, such as bone. In contrast to collagen, the mechanical strength of bone can be achieved by silk materials without the need to chemically crosslink the proteins, lowering the probability of cellular toxicity, inflammatory response, and other undesired effects [112]. A study involved loading silk scaffolds with aspirin and using controlled drug release to determine the rate of skull tissue regeneration has been conducted in mice [14]. Silk and silk-composite scaffolds were tested, both showing effective drug release and tissue regeneration in vivo.

### 5.2. Antibiotic Release

Antibiotics date back to the early 1940s. The development of penicillin during this time revolutionized the world of medicine. One major area of study is the development of antibiotics with less bacterial resistance. However, another area of focus, administration, must not be overlooked [15]. There are areas of the body, such as bone and eyes, to which it is harder to administer antibiotics. Drug-loaded protein materials can aid in administering to rather difficult areas. Due to their tunable properties, drug release can be controlled in a substance that can match the mechanical properties and stress of natural tissue in those environments.

Drug-loaded silk films, microspheres, and hydrogels have all been shown to release penicillin and ampicillin tests proving in vivo efficacy in a murine infected wound model. Silk nanoparticles, in particular, have been shown to have high effectiveness when used in conjunction with metallic implants [113]. Several issues can arise when metallic and metal-based implants are first inserted. These include bacterial infection, inflammation, and implant loosening [114]. Titanium is a typical metal used in metal and metallic implants. A recent study infused silk nanoparticles with the antibiotic gentamicin [114]. The particles were then coated a titanium surface in an in vitro study. Over an 18-day period, the drug-loaded silk nanoparticles showed more efficient bacteria inhibition when compared to pure titanium [113].

Collagen hydrogels are also applicable for the sustained delivery of antibiotics [15]. For example, patented formulations for collagen hydrogels has been used to substitute daily eye drop administration [15]. Antibiotics or other drugs were first solidified into eye drop gels. By controlling biodegradation rate of collagen network, the gel can remain until the necessary dosages have been administered [15].

### 5.3. Diabetes

Diabetes is a condition that affects the way one’s body reacts to insulin [115]. The most common forms of diabetes are type 1 and type 2. Type 1 diabetes indicates the pancreas’s inability to produce a sufficient amount of insulin. As a result, patients must administer external insulin frequently. Type 2 diabetes is the most common form of diabetes, affecting 85–90% of diabetes patients [115]. This type of diabetes indicates insulin resistance or reduced insulin secretion with insulin resistance [115]. By 2025, diabetes is predicted to have affected 280 million patients worldwide [115]. As such, advances in treating diabetes are currently under heavy research. Improving drug release while reducing the frequency of administrations through, for example, protein materials, is within the realm of possibility.

Insulin is the key to aiding diabetes patients in reducing blood glucose levels. For about 80 years, hypodermic injections of insulin have been the standard method of insulin delivery. However, insulin activity is lost in the blood within 2–3 h. Preparing insulin bonded with silk nanoparticles increased the half-life by about 2.5 times more than pure insulin [43]. Crystalline silk nanoparticles were covalently bonded to insulin, showing high recovery from insulin-silk systems (>90%) [43].

ELP depots can also be used for diabetes treatment and complications from diabetes [116]. Treatment can be observed without having the depot directly in the disease site. Drugs for type 2 diabetes, such as glucagon-like peptide 1 (GLP-1), have potential therapeutic effects but require many administrations [22]. Due to the relatively quick degradation of GLP-1, an ELP-GLP-1 fusion depot was fabricated to provide a long-term and sustained admission of the drug [22]. This medication can be genetically fused to ELP molecules with arginine residues before the proteolytic cleavage of the arginine residues release the GLP-1 peptide drugs.

### 5.4. Cancer Treatment

Cancer is a disease that affects the formation of cells. It causes out-of-control cell growth, resulting in tumor build-up. Cancer has become one of the leading causes of death since the turn of the century. Administration of cancer-fighting drugs is an attractive area of study. The nature of cancer typically requires frequent administration, numerous clinical visits, and multiple procedures for drug administration. As a result, studies have turned to proteins for alternatives to the regular visits and administrations. Elastin and silk-based materials show potential in remedying such issues.

ELPs have tunable phase transition (temperature control), making them ideal for local and sustained drug delivery [22]. Cancerous tumors, for example, require prolonged drug delivery for treatment. Therefore, ELP depots were used for radiation therapy of cancerous tumors [22]. Adjusting ELP properties such as injection concentration and composition further optimized the depot for cancer treatment.

Silk nanoparticles can also be loaded with drugs such as curcumin and injected locally to areas with cancer cells. In vitro testing, for example, showed the killing of breast cancer cells over an eight-day period using drug-loaded silk nanoparticles [28]. Another study used desolvation to produce silk nanoparticles [28]. These particles improved the delivery of doxorubicin, a drug commonly used in chemotherapy, to the cancerous site [28].

### 5.5. Other Potential Applications

#### 5.5.1. Neuroinflammation

Neuroinflammation is inflammation of the nervous tissue, resulting from brain injury, strokes, intracerebral hemorrhages, and other traumatic events [116]. Anti-inflammatory drugs are best administered locally. Using temperature-triggered ELP aggregation of nanoparticle depots can significantly decrease the loss of drug to circulation, optimizing the drug’s contact to the site of inflammation [22].

#### 5.5.2. Wound Healing

Surface wounds typically require disinfecting before covering with a bandage. Some injuries, however, require additional drugs for full treatment. Silk films have been tested for drug delivery and covering on skin surface [117]. In one study, silk and gelatin mixture was used in an in vitro test using rats [117]. Polyethylene glycol was added to the blend to reduce degradation speed. The result was promising, healing the wound in one week after the release of all of the drug loaded into the protein-composite film [117].

#### 5.5.3. Corneal Regeneration

Corneal disease is the leading cause of blindness. The only widely accepted and effective treatment method is keratoplasty, a transplant from a healthy, human donor. With the shortage of donor tissue, the increasing demand for corneal graft replacements have made allogenic and synthetic materials the gold standard [118]. Although allogenic materials have excellent mechanical and physiological properties, their applicability is limited by quality-donor corneal graft availability, immunological rejection, and the risk of carrying viruses [18]. To address the shortages, synthetic-based polymers were developed. They are usually easy to process, modify and fabricate into any shape or structure to satisfy the patients’ specifications. Nevertheless, synthetic keratoprostheses still require donor corneal tissue and can lead to other complications such as corneal melting, inflammation, necrosis, scarring, uveitis, and endophthalmitis [17]. To avoid the high costs of pre-transplant screening for transmissible pathogens and serious complications from a donor-derived infection in tissue banking, protein-based polymers, such as collagen, elastin, and silk proteins, can provide an excellent substitute for corneal regeneration [18]. To achieve key mechanical and physiological functions, Ghezzi et al. fabricated a three-dimensional (3D) silk culture system that closely resembled the native corneal stroma tissue [17]. As designed, the group showed seamless host-graft integration, cell organization and tissue regeneration in the stacked silk protein films after implantation. Protein-based polymer materials possess both physical and biofunctional properties needed to promote cell adhesion and cell proliferation as well as facilitate tissue repair.

## 6. Conclusions

Although the topic of controlled-release drug delivery is not new, advances in materials science have allowed for a diverse repertoire of new drug developments. Admission of such drugs can be achieved through various polymers and other materials. Synthetic polymers introduce the issue of biocompatibility, resulting in extensive testing to prove compatibility. Usage of various naturally occurring proteins and composites of such proteins show promise in improving drug delivery. Although biocompatibility must still be established, using naturally occurring proteins can minimize biocompatibility issues. Some drugs, for example, can also make use of the composition of the proteins used, binding to the amino acid components of the material.

This review focused on the properties and prospect of protein materials such as silk, collagen, elastin, and keratin. Each can be processed into various forms (microparticles, hydrogels, microneedles, etc.) to suit the application better. As research continues, more fabrication methods will arise, resulting in new diseases to be treated using proteins. Of course, there are still many challenges to be met. Some drugs, for example, may require development under high temperatures, which would denature proteins. In time, these challenges will be faced and overcome by fabricating thermally stable protein-composite materials. In time, development of other proteins with proven biomedical use can be adapted to drug-release applications. In time, the field will continue to grow, opening more avenues of research, more opportunities to fight disease, and more achievements in the area of science.

## Figures and Tables

**Table 1:** Biomedical applications of protein biomaterial and their structural design.

Material	Applications	Structural Design
Collagen	Engineering of cartilage, corneal, nerve, ocular, skin, and tendon/ligament tissues, surgical conduits, wound repair, integrated in a variety of composite materials to enhance favorable drug-delivery properties	Hydrogels [15,38,39,40], Films [17], Fibers [41]
Elastin	Controlled drug delivery, engineering of cartilage, liver, ocular, and vascular graft tissue, highly tunable thermoresponsive intracellular functionalized peptide drugs, wound healing applications	Hydrogels [42,43,44], Films [45,46], Fibers [47,48]
Keratin	Antibacterial, drug delivery, tissue engineering, trauma and medical devices, wound healing	Hydrogels [49,50,51,52], Films, [53], Fibers [54]
Resilin	Engineering of native vocal fold, cardiovascular, human cartilage tissues, protein-engineered bioactive materials to promote cell adhesion, degradation, growth factor delivery, and cell differentiation	Hydrogels [34,55,56,57], Nanoparticles [58]
Silk	Adhesive fillers, engineering of cartilage or load bearing tissues, wound dressing, enzyme immobilization, drug delivery	Hydrogels [59], Films [60], Microcapsules [61], Microparticles [62]
Zein	Biomineralization, controlled drug release, enhanced mechanical strength, microbial resistance, positive cell attachment and osteoblast growth	Films [63], Microspheres [64], Nanofibers [65], Nanoparticles [66]
